# Emergent Genome-Wide Control in Wildtype and Genetically Mutated Lipopolysaccarides-Stimulated Macrophages

**DOI:** 10.1371/journal.pone.0004905

**Published:** 2009-03-20

**Authors:** Masa Tsuchiya, Vincent Piras, Sangdun Choi, Shizuo Akira, Masaru Tomita, Alessandro Giuliani, Kumar Selvarajoo

**Affiliations:** 1 Institute for Advanced Biosciences, Keio University, Tsuruoka, Japan; 2 Systems Biology Program, School of Media and Governance, Keio University, Fujisawa, Japan; 3 Department of Molecular Science and Technology, Ajou University, Suwon, Korea; 4 Department of Host Defense, Research Institute for Microbial Diseases, Osaka University, Osaka, Japan; 5 Istituto Superiore di Sanita', Environment and Health Department, Rome, Italy; Institut Pasteur Korea, Republic of Korea

## Abstract

Large-scale gene expression studies have mainly focused on highly expressed and ‘discriminatory’ genes to decipher key regulatory processes. Biological responses are consequence of the concerted action of gene regulatory network, thus, limiting our attention to genes having the most significant variations is insufficient for a thorough understanding of emergent whole genome response. Here we comprehensively analyzed the temporal oligonucleotide microarray data of lipopolysaccharide (LPS) stimulated macrophages in 4 genotypes; wildtype, Myeloid Differentiation factor 88 (MyD88) knockout (KO), TIR-domain-containing adapter-inducing interferon-β (TRIF) KO and MyD88/TRIF double KO (DKO). Pearson correlations computed on the whole genome expression between different genotypes are extremely high (>0.98), indicating a strong co-regulation of the entire expression network. Further correlation analyses reveal genome-wide response is *biphasic*, i) *acute-stochastic* mode consisting of small number of sharply induced immune-related genes and ii) *collective* mode consisting of majority of weakly induced genes of diverse cellular processes which collectively adjust their expression level. Notably, temporal correlations of a small number of randomly selected genes from *collective* mode show scalability. Furthermore, in *collective* mode, the transition from large scatter in expression distributions for single ORFs to smooth linear lines emerges as an organizing principle when grouping of 50 ORFs and above. With this emergent behavior, the role of MyD88, TRIF and novel MyD88, TRIF-independent processes for gene induction can be linearly superposed to decipher quantitative whole genome differential control of transcriptional and mRNA decay machineries. Our work demonstrates genome-wide co-regulated responses subsequent to specific innate immune stimulus which have been largely neglected.

## Introduction

The innate immune system utilizes pattern-recognition receptors (PRRs), present in phagocytes such as macrophages and dendritic cells, to recognize pathogen associated molecular patterns (PAMPs), such as lipopolysaccarides (LPS). LPS, which is found on the outer membrane of Gram-negative bacteria, through the Toll-like receptor (TLR) 4, triggers a cascade of signaling events initiated mainly by the MyD88- and TRIF-dependent pathways. This activates a number of common transcription factors including activator protein (AP)-1, nuclear factor–κB (NF-κB) and interferon regulatory factors (IRF)-3. As a result, a number of cytokines such as IL-1β and TNF-α, and type I interferons such as IFN-α and IFN-β are produced. These proinflammatory mediators activate helper T-cells for the onset of acquired immune defense where foreign intruders are eliminated and immunological memory is created [1]–[5]. These processes happen at a multi-cellular level implying the coordinated activities of many different tissues. Immunological responses are self-limiting, highly orchestrated systemic processes that if not precisely controlled can lead to major illnesses such as autoimmune diseases, cardiovascular diseases and cancer [6]–[8].

Recent high throughput experimental technologies have enabled the comprehensive analysis of cellular response to a given stimulus. However, molecular immunology still largely follows the tradition of analyzing the snapshot of only a small number of specific statistically significant molecules' response. Although analyzing statistically significant genes can help in the explanation of ‘local’ specific response, to grasp the global regulatory processes requires the comprehensive understanding of genome-wide response [9].

A previous high throughput study on LPS-stimulated murine macrophages (in wildtype, MyD88 KO, TRIF KO, and MyD88/TRIF double (DKO)) focused on 148 highly expressed genes out of 22690 ORFs based on 3-fold expression increase and 100 expression unit cut-off from 0 to 4 h after LPS stimulation [10]. Although the study showed novel local insights of immune-related genes in different KOs, it did not show the capacity of LPS to induce pleiotropic biological processes not directly linked to immunity [11], [12]. To infer system-level emergent complexity, we re-investigated the same data without any biased expressions cut-off.

Building upon the accuracy and reliability of a correlation metrics based upon thousands of statistical units (genes, ORFs), we investigated temporal whole genome response to LPS stimulation in the above-mentioned 4 different genotypes of macrophages. In contrast to individually analyzing each microarray elements (ORFs), where weakly expressed ORFs usually has been considered to incur high noise-to-signal ratio, we analyzed the temporal expression changes of entire ORFs set considered as a whole. We confirmed LPS induces pleiotropic biological response and, additionally, found two characteristic response modes: *acute-stochastic* and *collective*. *Acute-stochastic* mode is largely immune-related response, while *collective* mode participates in diverse regulatory processes normally unrelated to immunity that transform the initial local response to the antigenic stimulus into a general state change of the cell. Overall, we show genome-wide differential response mainly occur through lowly expressed genes. This global LPS response has been previously neglected by biased gene-expression cut-offs. Our work can be used to understand both specific and global responses of biological systems.

## Results and Discussion

### Genome-wide invariance between wildtype, single and double KOs

**Figure 1:**
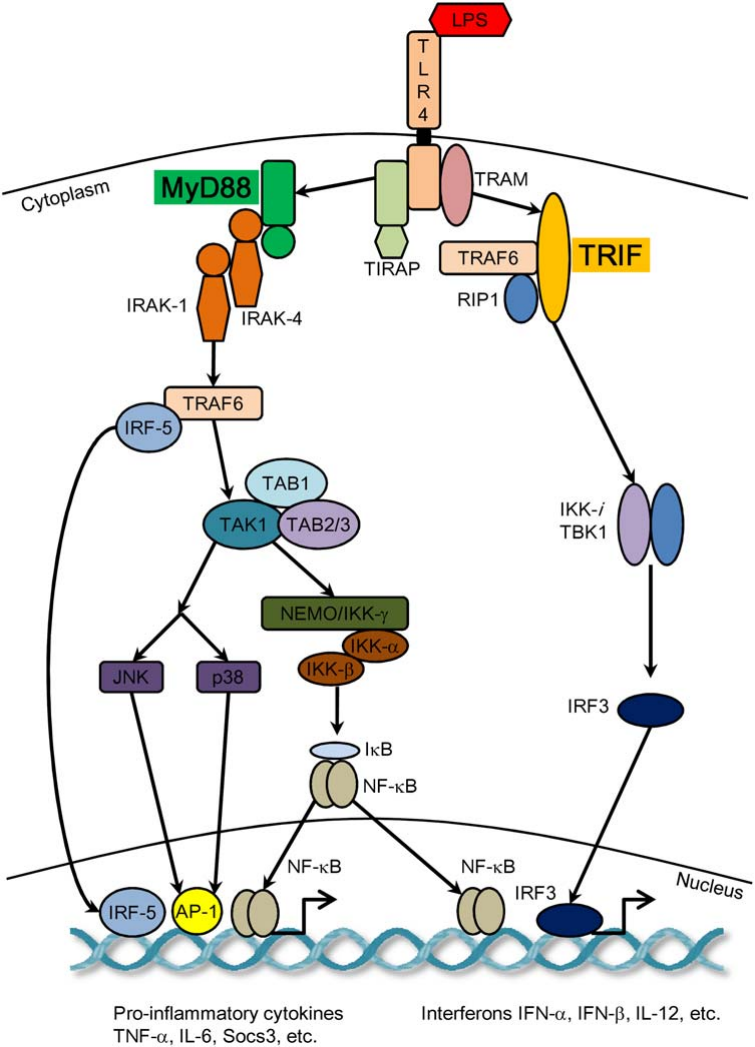
Simplified overview of LPS-induced signaling. LPS binds with TLR4 and activates transcription factors AP-1, NF-κB and IRF3 through MyD88- and TRIF- dependant pathways. This leads to the induction of proinflammatory cytokines and interferons. Figure modified from Akira *et al.* (2006).

**Figure 2:**
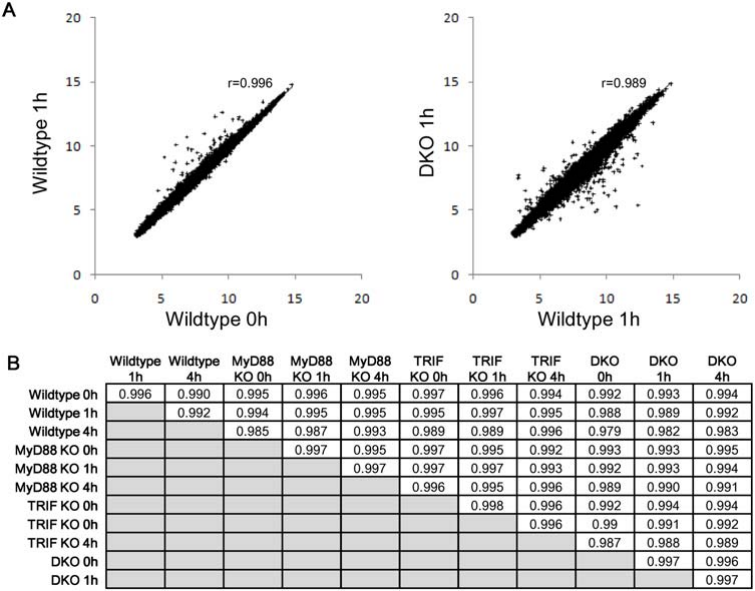
Genome-wide invariance between wildtype, single and double KOs. Highly correlated gene expressions between genotypes, and between time points. A) Left panel: wildtype 0 h (x-axis) vs. wildtype 1 h (y-axis), right panel: wildtype 1 h (x-axis) vs. DKO 1 h (y-axis). Other combinations of genotype and time points also show similar correlations (data not shown). Each point in the plot represents the expression of a single ORF. B) Whole genome Pearson correlations between samples.

LPS stimulates the MyD88- and TRIF-dependent pathways to activate the innate immune response (Figure 1). We evaluated the correlation structure of Affymetrix mouse expression data for wildtype, MyD88 KO, TRIF KO, DKO at 0, 1 and 4 hours (Methods and [10]). The analyses of only highly up- or down-regulated gene expressions ignoring lowly-expressed genes may not be sufficient to unravel genome-wide organizational principles [13]–[15]. To investigate the existence of such principles, we analyzed the whole genome cDNA microarray expression without any threshold cut-off. First, we performed Pearson correlation analysis on the entire genome expression vector. This ensemble property of the population of genes is a robust measure that is not biased by noise at the level of individual gene measurement [16], [17]. The whole genome correlations of the same cell-type between different genotypes are extremely high (Pearson *r* above 0.98, Figure 2A–B), indicating a strong common order parameter influencing the expression level of the entire genome, correspondent to the cell-type characterization [17]. High correlation between genotypes may suggest that technical noise of our microarray dataset is rather low [18], [19]. Furthermore, our results are coincident with the results obtained on erythroid cell lineages using the same metrics [20]. The presence of such invariant order spanning more than twenty-thousand elements (genes, ORFs) and around four orders of magnitude of expression levels is a signature of general order parameters organizing the entire cell regulation network. This organization is, in our opinion, a ‘fact of nature’ that, for its dimensions and invariance, asks for a deep thinking in analogy of what happened for other collective phenomena in physics (magnetism, laser coherence, super-fluid helium, hydrodynamic instabilities). Such strong invariance is imposed by the presence of a common attractor correspondent to the cell kind [16], [20], between all genotypes especially when we already know that MyD88 KO and DKO show significantly impaired proinflammatory responses [21]. Hence, to investigate specific proinflammatory and global LPS response, we compared the between genotypes Pearson similarities as computed both on the whole genome and on different extractions of gene subsets (random and immune-related).

### Temporal Pearson correlation reveals genotype differences

We adopted two measures to compare different genotypes by means of a Pearson correlation metrics: i) *auto-correlation*: Pearson *r* between 0 h (*t_0_*) and other time points (*t_1_*, *t_4_*) of the same genotype and ii) *cross-correlation*: Pearson *r* between wildtype and other genotypes at same time point (Methods). The *auto-correlation* analysis measures progressive response from *t_0_* for the same genotype, while the *cross-correlation* analysis measures the temporal difference from wildtype response.

**Figure 3:**
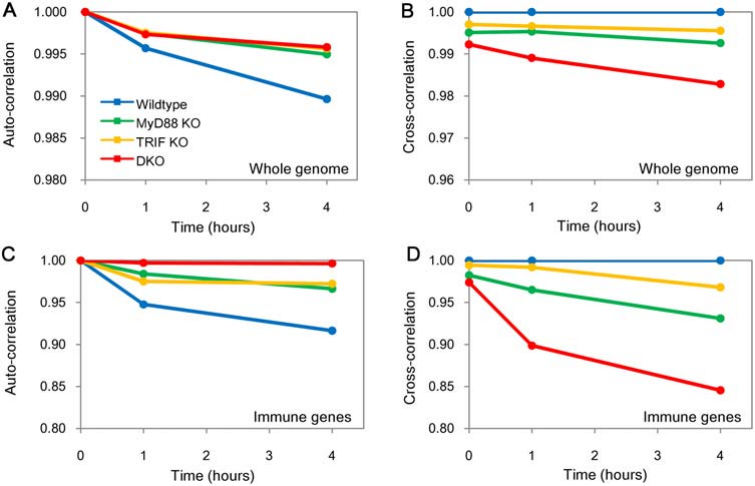
Temporal Pearson correlation reveals genotype differences. A) *Auto*- and B) *cross*-correlations for whole genome (22690 ORFs). C) *Auto*- and D) *cross*-correlations for immune-related genes. See maintext for details. Immune-related genes constitute 157 well-known genes induced during immune and inflammatory response (obtained from GenMAPP [20]).

The whole genome *auto-correlation* of all genotypes shows progressive response, correspondent to a progressive displacement of correlation from unity, to LPS stimulation using RMA normalized data (Figure 3A and Methods). We further checked the consistency of this result using another normalization process, MAS5 data (Figure S1A). Wildtype response follows distinctly different trend from MyD88 KO, TRIF KO and DKO that in turn are remarkably similar to each other. These results show i) 1% of *auto-correlations* variability is sufficient to discriminate different genotypes, and ii) the similar global gene expression behavior between single KOs and DKO suggests DKO possesses LPS response, too (see also section ‘Emergence of regulatory signature from scattered expressions in all genotypes’ for further proof of DKO response).

To find the source for genome-wide response similarity between DKO and single KOs, we calculated the *cross-correlation* for all genotypes. *Cross-correlation* shows the response of TRIF KO is the most similar to wildtype, followed by MyD88 KO and DKO (Figure 3B and Figure S1B). Although the *auto-correlations* are similar, the different *cross-correlations* of other genotypes, with wildtype as a reference, show that the gene expression responses between genotypes are distinct from one another. We find these differences originate from differential activation machinery of gene expressions (see section ‘Deciphering gene regulatory mechanisms from emergent signature’).

Since LPS is a well known inducer of immune response, we next, specifically focused on the *auto-correlation*s of 157 immune-related genes (according to GenMAPP [22], Table S1). By this, we shift the focus from the ‘whole-genome’ response to the ‘local immune-related’ response of the system. The result shows that DKO has a flat profile (as expected, almost perfect correlation between different times is observed due to the lack of any classical immune response to LPS), followed by TRIF KO and MyD88 KO displaying a linear displacement in time from unity correlation but to a lesser extent than wildtype, pointing to a diminished immune response of single KO with respect to wildtype. (Figure 3C and Figure S1C). These results are consistent with current literature which suggests that MyD88 is key for LPS stimulation and consequently, for DKO, no activation of immune response is expected to occur [23], [24].

The *cross-correlations* of immune-related genes show TRIF KO was closest to wildtype response, followed by MyD88 KO, while DKO was the farthest (Figure 3D and Figure S1D). Based on these results, we concur that the i) 157 immune-related genes are dependent on both MyD88 and TRIF, and that they are mainly activated by the MyD88-dependent pathway, ii) MyD88 and TRIF cannot be considered to be acting along the same pathway, otherwise single and DKO's *cross-correlation* should be equal and overlapping and iii) MyD88- and TRIF-dependent pathways are not completely independent, otherwise DKO's distance from unity in terms of *cross-correlation* would be equal to a composition of MyD88 KO and TRIF KO individual values. These results imply the existence of synergized action between the MyD88- and TRIF- dependent pathways because the sum of single KO responses is different from DKO response [25], [26] (see *MT* in ‘Deciphering gene regulatory mechanisms from the emergent signature’).

In summary, even though we observed genome-wide strong invariance between genotypes, temporal correlation analyses reveal the difference between them. The temporal whole genome (global) response and immune (local-specific) response show distinct profiles [27]. Thus, we pondered whether there are organization principles that distinguish such different modes of response. To uncover, we next investigated Pearson *r* of all genotypes between 0–1 h by selectively removing ORFs from each genotype. This procedure will allow us to individualize the different contributors to the whole genome behavior.

### Biphasic *acute-stochastic* and *collective* modes of LPS response

**Figure 4:**
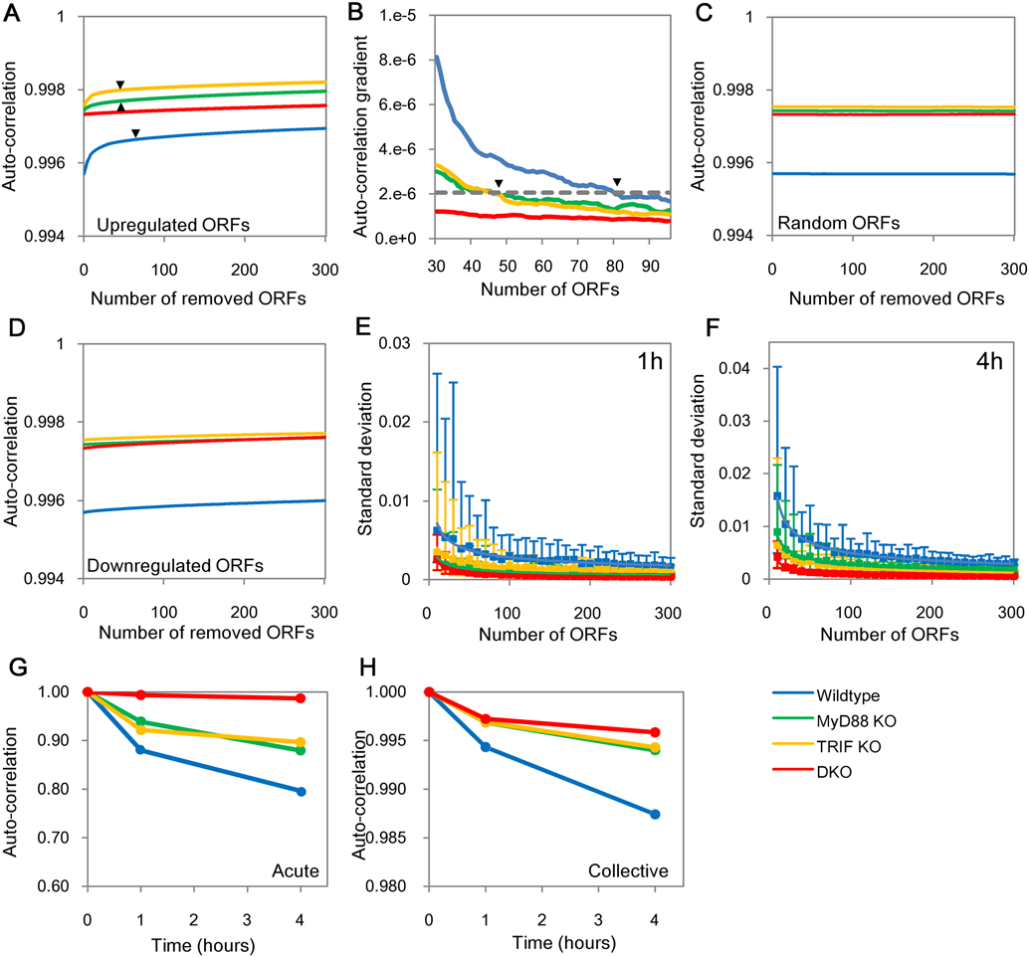
LPS induces biphasic *acute-stochastic* and *collective* modes of response in wildtype and single KOs but not DKO. A) *Auto-correlation* profiles of all genotypes when removing one by one up to 300 ORFs highest upregulated ORFs from 0 to 1 hr (in terms of expression change: Δ*x* = *x*(1 h)−*x*(0 h)). B) Plot of average *auto-correlations* slopes of 0–1 h in A). Since DKO possesses only *collective* mode, we used average slope of DKO curve at *N* = 10 to distinguish biphasic transition (dotted gray line). Wildtype and single KO cross this slope at about *N* = 80 and *N* = 50 ORFs, respectively. This *biphasic* transition point further suggests *collective* mode. No biphasic behavior was found for C) randomly selected or D) downregulated ORFs. Confirmation of *collective* mode: E) Standard deviation (SD) of *auto-correlation* (0–1 h) of groups of randomly chosen ORFs in steps of 10 up to 300 from whole genome. Each point represents average SD of 30 groups, error bars represent highest and lowest SD. F) As in E) for 1–4 h *auto-correlation*. G) *Auto-correlations* of 80 highly expressed ORFs representing *acute-stochastic* mode in the wildtype. H) Average *auto-correlations* of 30 extractions of 80 randomly chosen ORFs in the wildtype *collective* mode.

We analyzed the change (i.e., difference of expression) of response based on Pearson *r* of all genotypes by removing highest up- and down-regulated ORFs (one by one up to 300 ORFs) between 0 to 1 hr. For removing highest up-regulated ORFs, a *biphasic* (hyperbolic) phenomenon emerges in wildtype and single KOs but not in DKO (Figure 4A). The gradient of curves is steep up to the removal of about 80 ORFs in wildtype and 50 ORFs in both single KOs after which the slope gentles. In contrast, in DKO, only the gentle gradient exists (Figure 4B). As control, we next compared randomly removed ORFs in similar steps and found Pearson *r* (0–1 h) of all genotypes does not change noticeably (Figure 4C). This result points to a transition in the response: a small fraction of ‘acute’ responding ORFs, and the majority of ORFs responding ‘weakly’. Notably, for downregulated ORFs, *biphasic* response is not observed for any genotype (Figure 4D).

To investigate further, we evaluated the standard deviation of Pearson *r* (0–1 h) for randomly chosen ORFs in steps of 10 up to 300 (with each selection repeated 30 times) from whole genome and measured the standard deviation (SD) of *auto-correlation*. We notice that the mean value of SD of *auto-correlation* decreases with number of ORFs selected (Figure 4E). Notably, at around 80 ORFs for wildtype (and 50 for single KOs) the *auto-correlation* transits to a flat trend (DKO, however, did not show any transition). The amplitude of SD of *auto-correlation* is large for less than 80 ORFs for wildtype (and around 50 for single KOs) and small otherwise (Figure 4E). These results reveal the coincidence of *biphasic* transition at around 50 ORFs. Therefore, we can consider acute response as *acute-stochastic* mode (high SD) and the weak response as *collective* mode (low SD) [27]. We also found *biphasic* response for *auto-correlations* between 1–4 h (Figure 4F).

Investigating further the temporal response of *acute-stochastic* and *collective* modes we observed *auto*- and *cross-correlation* profiles of *acute-stochastic* mode is similar to immune-related genes whereas the average response of randomly selected 80 ORFs in *collective* mode is scaled to genome-wide response (Figure 4G–H, and Figure 3A), thus demonstrating that *collective* mode is scalable. This implies the *collective* mode corresponds to a global, a-specific adjustment of the expression network that can be appreciated even in the case of a sufficiently populated random sample of genes. The *collective* mode can be considered as a sort of ‘mean field’ encompassing the entire genome expression and needs a sample of at least 80 ORFs to be reliably detected.

### Emergence of regulatory signature from scattered expressions in all genotypes

**Figure 5:**
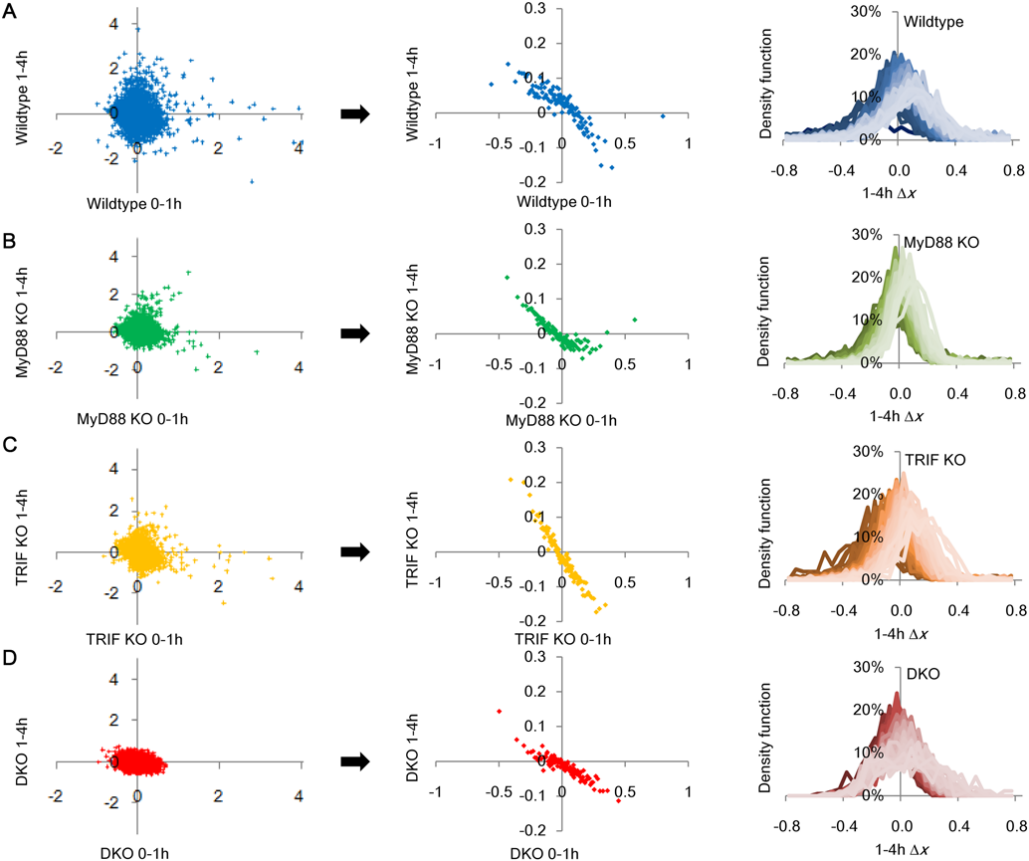
Emergence of regulatory signature from scattered expressions. Genome-wide expression changes (Δ*x*) between time points, 0–1 h (x-axis) *vs.* 1–4 h (y-axis) for single ORF (left panels) in A) wildtype, B) MyD88 KO, C) TRIF KO), and D) DKO. Center panels: corresponding plots for group of 200 ORFs sorted by their 0–1 h expression change (x-axis). Each point represents the average of Δ*x* for 200 ORFs. Right panels: Gaussian distribution of Δ*x* for 1–4 h. Superposed profiles (lighter color for increasing upregulated groups and darker color for increasing downregulated groups) represent density distribution (Gaussian) of each group of 200 ORFs sorted from highest to lowest for 0–1 h in A) wildtype, B) MyD88 KO, C) TRIF KO and D) DKO. *x*-axis represents Δ*x* for 1–4 h and *y*-axis represents the density of ORFs.

To understand the temporal progress of *biphasic* response, we investigated the changes of whole genome expression from early (0–1 h) to late (1–4 h) in each genotype. We plotted the expression change (Δ*x*) of single ORFs (0–1 h for *x*-axis and 1–4 h for *y*-axis) (Figure 5A–D, left panels) and the average value of groups of ORFs (10, 50, 80, and 200 ORFs) (0–1 h for *x*-axis and 1–4 h for *y*-axis) sorted from highest to lowest expressions (Figure 5A–D, center panels for 200 ORFs and Figure S2). Remarkably, we observed the transition from large scatter in expression distributions around the origin for single ORFs to smooth lines for group of 50 ORFs onwards for all genotypes. This is due to the fact that grouping of expression distribution for 50 ORFs or above forms Gaussian distribution and the average value of each group remarkably follows linear line (Figure S3A and see ‘Linear regressions analyses’ in Methods); the fact average values follow linear lines reveals the emergence of regulatory signature working at the level of groups of genes. Furthermore, the fluctuations on Gaussian distribution reduce as we increase the grouping size (Figure S3A–C). From these, we observe genes upregulated at early time points were downregulated at later time points and vice-versa, through switching in gene regulatory circuits. Biologically, upregulated ORFs in early signaling, when transcription rate is faster than mRNA decay, are downregulated by late signaling, when mRNA decay is prevalent. The vice versa occurs for downregulated ORFs. Notably, this switching behavior is also observed for DKO, thus reinforcing that DKO possess genome-wide LPS response through MyD88- and TRIF- independent manner.

### Deciphering gene regulatory mechanisms from emergent signature

**Figure 6:**
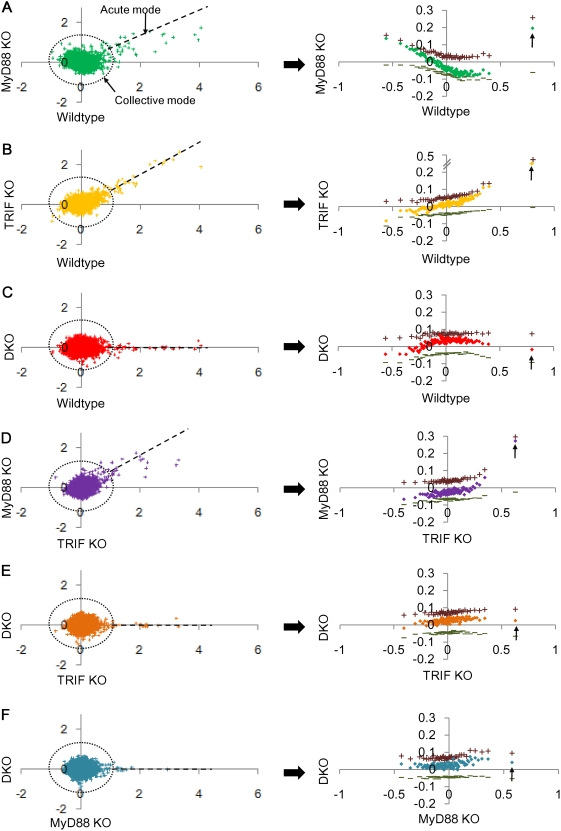
Large scatter in *collective* mode and linear distribution in *acute-stochastic* mode. Genome-wide single ORFs (left panels) expression changes (Δ*x*) for 0–1 h between genotypes: wildtype *vs.* A) MyD88 KO, B) TRIF KO, C) DKO; TRIF KO *vs.* D) MyD88 KO and E) DKO; F) MyD88 KO *vs.* DKO. Right panels: corresponding plots for group of 200 ORFs, sorted by their expression change in the corresponding genotype (x-axis). + and − indicate average of expression change of the upregulated and downregulated ORFs in each group, respectively. Arrows indicate groups containing the *acute-stochastic* mode.

We observed earlier from *auto* and *cross-correlations* that the gene expression responses between genotypes are distinct from one another. To understand this in depths, we compared genome-wide expression changes between genotypes for 0–1 h, total of 6 combinations (Wildtype *vs.* MyD88 KO, Wildtype *vs.* TRIF KO, Wildtype *vs.* DKO, TRIF KO *vs.* MyD88 KO, TRIF KO *vs.* DKO and MyD88 KO *vs.* DKO) (Figure 6A–F). We plotted the expression change (Δ*x*) of single ORF as well as taking the average value of each group of ORFs sorted from highest to lowest expressions. We observed i) large scatter in expression distributions around the origin for ORFs in the *collective* mode and ii) linear expression distribution of ORFs in the *acute-stochastic* mode (Figure 6A–F, left panels). These results further confirm biphasic response of LPS stimulation.

For the *collective* mode, we found the distribution of averages from expression change (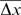) in each group follows transition from scatter to smooth lines for group of 50 ORFs onwards for all genotypes (Figure 6A–F, right panels & Figure S4), revealing the emergence of genome-wide control in wildtype and all KOs. This is due to the fact that when we group ORFs of 50 and above, the distribution of Δ*x* becomes Gaussian and the mean values of distribution follow linearity in the same way as mentioned in previous section. Another observation is that DKO emergent signature is invariant from single KOs (Figure 6E–F, right panels). This further suggests that the contribution from DKO is independent from MyD88 and TRIF and derives from an unknown source (*U*). Thus, the emergent signature as smooth line can be used to decipher quantitative genome-wide differential control of transcriptional (Tr) and mRNA decay machineries (Dm) by considering individual roles of MyD88 (*M*), TRIF (*T*), their collective/synergistic roles (*MT*) and MyD88/TRIF-independent processes (*U*) for gene induction, i.e., wildtype = *M*+*T*+*MT*+*U*, MyD88 KO = *T*+*U*, TRIF KO = *M*+*U* and DKO = *U*. This linear superposition model (resembling analysis of variance scheme) cannot be applicable to analyze individual genes. However, for understanding averaging behaviors found in each genotype, it can help to shed light on overall control mechanisms of LPS stimulation.

Wildtype upregulated ORFs in *acute-stochastic* mode (WT_a_^+^), are constituted of a single group of 80 ORFs whose average expression change in wildtype (*M*+*T*+*MT*+*U*) is 1.22, MyD88 KO (*T*+*U*) is 0.46, TRIF KO (*M*+*U*) is 0.80 and DKO (*U*) is 0. Hence, the relative contribution of each signaling to the activation of *acute-stochastic* mode ORFs is determined: *M* = 0.80, *T* = 0.46, *MT* = −0.04, *U* = 0. From these values, MyD88-dependant (*M*) and TRIF-dependant (*T*) pathways are important for transcription while MyD88 & TRIF-dependent synergized processes (*MT*) are insignificant (in contrast to *collective* mode, see below). Furthermore, unknown processes (*U*) are not indicated for *acute-stochastic* mode. Using DAVID analysis platform [28], the majority of biological processes of *acute-stochastic* mode is related to immune system, defense response, inflammatory response, etc., specifically activated by *M* and *T* (Table 1, *p*<0.05).

**Table 1:** Biological processes enriched in wildtype upregulated *acute-stochastic* mode.

Biological processes (GO) and *pathways* (KEGG)	# genes	*p*-value
inflammatory response	13	5.6.E-06
response to wounding	13	9.9.E-05
defense response	14	2.9.E-04
response to stress	16	3.7.E-03
response to external stimulus	13	3.8.E-03
immune system process	14	2.8.E-02
immune response	11	3.6.E-02
response to stimulus	21	4.6.E-02
*Cytokine-cytokine receptor interaction*	9	2.5.E-02

**Figure 7:**
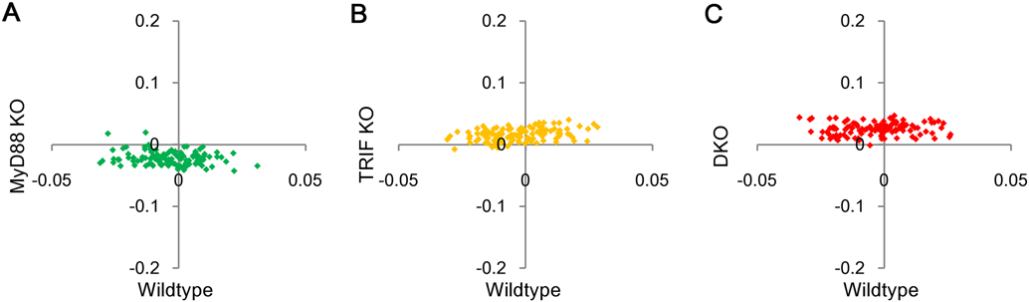
Global shifts in each genotype distribution. Genome-wide expression changes (Δ*x*) for groups of 200 randomly chosen ORFs between genotypes for 0–1 h: wildtype *vs.* A) MyD88 KO, B) TRIF KO, C) DKO.

Next, we compared groups of upregulated ORFs in the *collective* mode of wildtype (WT_c_^+^) (*x*-axis) with MyD88 KO and DKO (*y*-axes) (Figure 6A and C, right panels). Unlike *acute-stochastic* mode, *collective* mode possesses average points in plot which follows flat profiles. This could be due to the equilibrium of transcriptional and mRNA decay machineries or shift in experimental or normalization process. To investigate this statistically, we selected grouping of equal number of random ORFs and assessed their average expression change in wildtype against all genotypes. Flat profiles were observed with *M*+*T*+*MT*+*U* = 0 (wildtype), *T*+*U* = −0.024 (MyD88 KO), *M*+*U* = 0.013 (TRIF KO) and *U* = 0.024 (DKO) (Figure 7A–C). Since random sampling displays genome-wide constant shift in MyD88 KO, TRIF KO and DKO, these should be due to experimental shift or normalization process. Thus, we eliminated these shifts from MyD88 KO, TRIF KO and DKO contributions.

**Figure 8:**
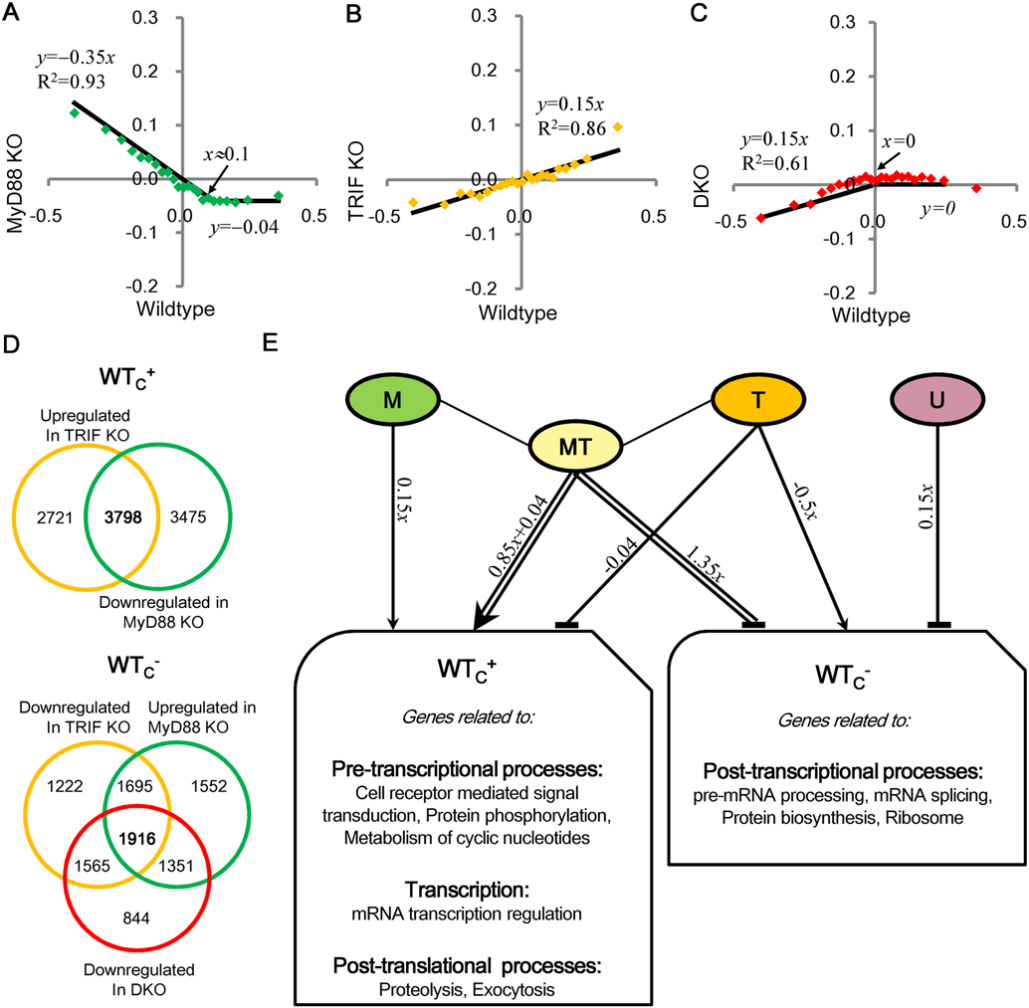
Deciphering gene regulatory mechanisms from the emergent signature. Linear regressions of genome-wide expression changes (Δ*x*) between genotypes after the removal of global shifts in Figure 7: A) wildtype vs. MyD88 KO, B) wildtype vs. TRIF KO, C) wildtype vs. DKO, for group of 1000 ORFs, sorted from highest to lowest expressions change in wildtype. Each point represents the average expression changes of 1000 ORFs (see Methods). Distributions are approximated to linear equations represented by *y* = *αx*+*β* (main text) so as to obtain best fit. Flat distributions are fitted to the average of their points (*R* ^2^ = 0). D) Gene clustering. The 3798 ORFs upregulated in TRIF KO and downregulated in MyD88 KO for WT_c_^+^ (top panel) and the 1916 ORFs downregulated in TRIF KO and DKO, and upregulated in MyD88 KO for WT_c_^−^ (bottom panel) were selected to determine biological processes regulated in whole genome (see maintext). E) Individual effects of *M*, *T*, *MT* and *U* on the biological processes regulated in *collective* mode of WT_c_^+^and WT_c_^−^. Arrows indicate activation through dominant transcription and T-shaped lines indicate repression through dominant mRNA decay.

In general, the emergent smooth lines in *collective* mode can be presented by *y* = *αx*+*β* (Figure 8A–C) where slope *α* represents net outcome between transcriptional and mRNA decay machineries on average expression change and y-axis interception, *β*, implies contribution from the equilibrium state of transcriptional and mRNA decay machineries. Note that we ignore analysis around the origin of axes, where the densely distributed average points results in overlapping of their Gaussian distributions between genotypes.

For WT_c_^+^, we observed same flat profile for DKO in both WT^+^ and randomly selected wildtype ORFs, which indicates that WT^+^ in DKO possess no response, i.e., *U* = 0 (Figure 8A and C, *x*>0). The flat response in DKO stems from different set of upregulated ORFs from WT^+^, confirming that DKO response (*U*) is independent of MyD88 and TRIF. From all genotypes' plot, we obtain *M*+*T*+*MT* = *x*, *T* = −0.04 and *M* = 0.15*x*, thus, *MT* = 0.85*x*+0.04 (Figure 8A–C, *x*>0). This result points to i) MyD88 & TRIF-dependent processes (*MT*) show synergized activation of genes through transcription, i.e., Tr≫Dm, since *MT* possesses dominant positive slope, ii) MyD88-dependent pathway (*M*) also activates these genes but to a lower extent, i.e., Tr>Dm, since *M* possesses smaller positive slope, iii) TRIF-dependent pathway (*T*) shows the equilibrium of transcriptional and mRNA decay machineries i.e., Tr–Dm = constant, since non-zero flat response between positive (transcriptional) and negative (mRNA decay) contribution.

For wildtype downregulated ORFs (WT_c_^−^), we obtained: *M*+*T*+*MT*+*U* = *x* (Note: *x*<0 for WT_c_^−^), *T*+*U* = −0.35*x*, *M*+*U* = 0.15*x*, *U* = 0.15*x*; thus, *M* = 0, *T* = −0.5*x*, *MT* = 1.35*x* and *U* = 0.15*x* (Figure 8A–C, *x*<0). This result points to i) MyD88 & TRIF-dependent (*MT*) pathways mainly show synergized repression of genes through mRNA decay, i.e., Tr≪Dm; ii) TRIF-dependent (*T*) pathways can activate the transcription of the same processes as *MT*, i.e., Tr>Dm; iii) *M* has no regulatory role for wildtype downregulated ORFs and iv) unknown processes (*U*) can weakly repress the same genes through decay machinery, i.e., Dm>Tr.

To determine biological processes regulated by the whole genome, we selected genes satisfying upregulation in TRIF KO and downregulation in MyD88 KO for WT_c_^+^ and genes satisfying downregulation in TRIF KO and DKO, and upregulation in MyD88 KO for WT_c_^−^, due to the observed emergent linearity (Figure 8D). We obtained 3798 out of 11793 ORFs for WT_c_^+^ and 1916 out of 10897 ORFs for WT_c_^−^. Next, combining WT_c_^+^ and WT_c_^−^ ORFs, and using DAVID platform analysis, we retained the biological processes of genes with significant enrichment (Tables 2 and 3, *p*<0.05).

**Table 2:** Biological processes enriched in the wildtype upregulated *collective* mode.

Biological processes (Panther) and *pathways* (KEGG)	# genes	*p*-value
Developmental processes	445	8.3.E-06
mRNA transcription regulation	1686	5.9.E-06
Cell structure	667	7.0.E-05
mRNA transcription	1044	3.1.E-04
Signal transduction	747	5.4.E-04
Proteolysis	969	1.9.E-03
*Melanogenesis*	*43*	*3.9.E-02*
Cation transport	776	6.0.E-03
Oxidative phosphorylation	287	7.3.E-03
*Cytokine-cytokine receptor interaction*	*86*	*4.0.E-02*
Neurogenesis	231	1.9.E-02
*Toll-like receptor signaling pathway*	*42*	*4.5.E-02*
Blood circulation and gas exchange	44	2.5.E-02
Cell structure and motility	309	2.3.E-02
*Focal adhesion*	*69*	*4.4.E-02*
Cell communication	195	2.4.E-02
*Prostate cancer*	*37*	*4.7.E-02*
Ion transport	445	2.7.E-02
*ErbB signaling pathway*	*33*	*4.5.E-02*
B-cell- and antibody-mediated immunity	104	3.2.E-02
Metabolism of cyclic nucleotides	99	3.4.E-02
Neuronal activities	82	3.2.E-02
Complement-mediated immunity	93	3.2.E-02
Cell surface receptor mediated signal transduction	428	3.4.E-02
Protein phosphorylation	449	3.9.E-02
Ectoderm development	122	4.4.E-02
Exocytosis	72	4.9.E-02

**Table 3:** Biological processes enriched in the wildtype downregulated *collective* mode.

Biological processes (Panther) and *pathways* (KEGG)	# genes	*p*-value
*Oxidative phosphorylation*	*54*	*1.2.E-16*
*Ribosome*	*37*	*1.1.E-09*
Protein biosynthesis	266	1.8.E-07
mRNA splicing	568	4.2.E-03
*Histidine metabolism*	*15*	*4.7.E-03*
*Proteasome*	*13*	*1.7.E-02*
Other metabolism	324	3.8.E-02
Pre-mRNA processing	363	3.0.E-02
*Valine*, *leucine and isoleucine degradation*	*15*	*3.4.E-02*
Protein metabolism and modification	305	4.7.E-02

We observed biological processes related to immunity (*Cytokine-cytokine receptor interaction*, *Toll-like receptor signaling pathway*, *etc.*) were upregulated in WT_c_^+^, which indicates immune-related genes are not restricted to the *acute-stochastic* mode alone (Table 2). Predominantly, however, in WT_c_^+^, a) pre-transcriptional and transcription-related genes, such as genes related to *cell surface receptor mediated signal transduction* (including *Protein phosphorylation* required for signal transduction and *Metabolism of cyclic nucleotides* used for kinase activities), and *mRNA transcription regulation* and b) genes related to post-translational processes (*Proteolysis*, *Exocytosis*, etc.) were upregulated (Table 2). In WT_c_^−^, c) genes related to post-transcriptional processes, (*Pre-mRNA processing*, *mRNA splicing*, *Protein biosynthesis*, *Ribosome*, etc.) were downregulated (Table 3). We therefore hypothesize that wildtype cells prepare for secondary immune activation (possibly through cytokine receptors) by upregulating signaling and transcription processes (Figure 8E). To self-regulate the secondary immune response, post-transcriptional processes (mRNA splicing and translation) are repressed. Interestingly, we observe, from Figure 8E, a complete reverse (switching) behavior in MyD88 KO where only T and U are active, e.g. pre-transcriptional processes and transcription become downregulated while post transcriptional processes are upregulated. This suggests MyD88 KO cells compensate lack of activation (transcription) by enhancing post-transcriptional processes through TRIF-dependent (*T*) pathways [29]. These findings seem to indicate that MyD88 is a key regulator in *collective* mode.

In summary, we observe differential activation of group of ORFs between genotypes (Table S2). From these results, we obtained the individual effects of *M*, *T*, *MT* and *U* on a genome-wide scale (Figure 8E). This shows genome-wide differential activation machinery of biological processes. Unlike *acute-stochastic* mode which is dedicated to immune system, *collective* mode may also participate in diverse regulatory processes; to prepare the cell for activation (signal transduction), prevent over expression of genes (regulation of post-transcriptional processes), and compensate lack of activation in KO conditions (enhancement of genes related to post-transcription when transcription process is lacking).

### Conclusion

In this report, we found quantitative genome-wide differential control of transcriptional and mRNA decay machineries through signaling processes superimposing over the general strong co-regulation of expression levels that is largely invariant between genotypes and related to the global expression attractor correspondent to the specific cell type [15], [20]. Moreover, each genotype, except DKO, possess two modes of responses; *acute-stochastic* (small number of immune-related genes) and *collective* modes (rest of ORFs). The *collective* mode, which consists of myriad cellular processes, is often ignored in most analyses as they are made of ORFs displaying small expression changes in time and hence cannot be captured if high cut-off thresholds (e.g. 3-fold) are used. Also in *collective* mode, for all genotypes, we notice scalable response and emergent linear behavior arise when ORFs are grouped. Other manifestations of such collective behavior, arising from the functional relations between gene expressions, were observed in terms of coordinated genome-wide expression waves [30], [31]. The transition from scatter to linearity was observed in the distribution of whole genome expression when grouping of 50 ORFs and above. Notably similar transition occurs for the distribution of single gene expression of cells in culture when the intrinsic (uncorrelated) noise becomes low [32]. Thus, our work also reveals the regulation by correlations in gene expression fluctuations [33]. However, it is important to stress that our data refers to large ensembles of cells, unlike single cell measurements, and thus exact discrimination between intrinsic and extrinsic noise sources cannot be performed in a similar manner [32], [33]. Nevertheless, the strong invariance between different conditions of the same cell-type, can be considered as a sort of dynamical attractor encompassing the entire transcriptome, reflecting hidden genome-wide differential regulations [27]. Understanding the link between the ordered behaviors observed for i) single gene expression when intrinsic noise is low [33] and ii) genome-wide conditions, promises to be a very fruitful future direction.

The discovery of two modes of response has also been shown recently for protein dynamics to a drug perturbation where a rapid translocation of specific proteins and a slower, wide-ranging temporal wave of protein degradation and accumulation occurred [34]. Our work points to the presence of a highly-ordered, coordinated, genome-wide expression dynamics of LPS stimulation, thereby requesting the need to consider global phenomena when interpreting immune response. In general, the consideration of the general rearrangement of the entire expression network after a specific stimulus, with the consequent activation of functions not directly linked to the original perturbation, could be the basis for rationalizing the onset of unexpected side-effects after drug treatments.

## Materials and Methods

### Biological datasets

We re-analyzed microarray data obtained from time-series experiments (0, 1, and 4 hours) performed on peritoneal macrophages from wildtype, MyD88 KO, TRIF KO, and MyD88/TRIF DKO mice treated with 100 ng/ml of LPS (*Salmonella Minnesota* Re595, Sigma) [10]. Affymetrix mouse expression array A430 microarray chips were used for gene expression detection. The microarray dataset obtained from these experiments contains expression levels for 22690 Affymetrix probe set IDs. We reprocessed our Affymetrix microarray chip data using Robust Multichip Average (RMA) for further background adjustment and to reduce false positives of our Affymetrix microarray chip [35]–[37]. The complete experimental details can be found in Hirotani *et al* [10].

### Statistical Analysis

#### *Auto*- and *cross-correlation* analysis for interpreting LPS response

To investigate the correlation between any two expression vectors, ***x*** and ***y*** with *n* ( = 22690) dimension and mean values of expressions 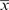 and 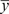, we calculate their mutual Pearson, 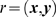 by
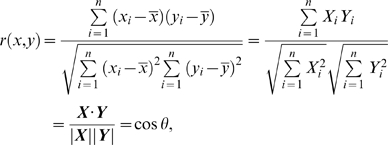
(1)where 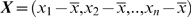, 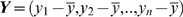 and *θ* is angle between two expression vectors. Geometrically, Eq. 1 shows the correlation coefficient, *r*(*x*,*y*), can be viewed as the cosine of the angle (*θ*) on *n*-dimensional space between the two vectors of data representing a measure of response. However, when *θ* = 0 (i.e., *r* = 1), generally ***X*** = α***Y*** (α>0). In the case when α = 1 i.e., ***X*** = ***Y***, this implies ***X*** and ***Y*** has the same response, otherwise, ***X*** and ***Y*** have different but *globally proportional* response.

We extend the Pearson correlation analysis to measure global temporal gene expression response to a given stimulation, comparing Pearson correlation between 0 h (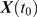) and all time points (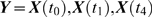) at 0 h, 1 h, 4 h of the same sample vectors, *auto-correlation*. Therefore, the *auto-correlation* profiles measures progressive divergence of expression from *t_0_* for each genotype in terms of decreasing correlation in time, if response of LPS stimulation occurs. Since Pearson correlations of whole genome for all condition are close to one (i.e., *θ*≅0), we need to distinguish whether α = 1 or not. We add 0 h vector elements of ***X*** into both ***X*** and ***Y***, resulting in 2*n*-dimension; ***X*** = (***X***(*t*_0_), ***X***(*t*_0_)) and ***Y*** = (***X***(*t*), ***X***(*t*_0_)). If ***X***(*t*) = ***X***(*t*_0_), that is, no response, then 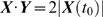, 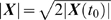 and 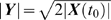, thus, *auto-correlation* = 1 (*θ* = 0). On the other hand, if ***X***(*t*) = α ***X***(*t*_0_) (α≠1 & α>0), *auto-correlation*, *r*≠1 (i.e. *θ*≠0). Biologically, *auto-correlation* with control profiles will show progressive divergence from 0 h expression for each genotype if dynamic response to LPS exists,.

Similarly to *auto-correlation*, *cross-correlation* is a temporal Pearson correlation measure. However, instead of measuring between the same genotypes with different time points, *cross-correlation* measures between different genotypes from wildtype at the same time points.

### Linear regressions analyses

To obtain reliable linear regressions in the analysis of expression change distributions (Figure 8A–C) for the *collective* mode, we determined the minimal number of ORFs (*N*) per group, to observe the formation of a Gaussian distribution centering on the average expression change of the group of ORFs in all genotypes. Shapiro-Wilk test was performed to assess the normality of the distribution of expression changes in each group of *N* (0<*N*<2000 by step of 10) ORFs, which provides a statistic *W* coupled with *p*-value of the data (Higher *W* and lower *p*-values for normally distributed data). The average of *p*-values computed were lower than 0.1 (2-tailed) for groups of about N>300 for MyD88 KO distribution, N>800 for TRIF KO and N>1000 for DKO (Figure S5). We therefore used *N* = 1000 to determine the linear regressions of expression changes distributions in *collective* mode.

### Functional enrichment analyses

DAVID functional annotation platform [28] was used to identify the functional categories (Gene Ontology (GO) [38], Panther gene classification [39] or KEGG pathways [40]) enriched in groups of ORFs. Among the 22690 ORFs, 10264 genes with annotations in Gene Ontology, 13824 genes with annotations in Panther gene classification, and 3778 genes with annotations in KEGG pathways were identified. To evaluate functional category enrichment under control of False Discovery Rate, Benjamini-Hochberg adjusted *p*-values were obtained for each term (GO, Panther, KEGG), and terms scoring *p*-values<0.05 were retained.

## Supporting Information

Table S1 List of immune-related genes. List of 157 immune-related genes selected from GenMAPP used for analysis.
(0.16 MB DOC)

Table S2 Differential activation of groups of ORFs between genotypes. Biological processes (Panther) and *pathways* (KEGG) enriched (*p*<0.05, Fisher-exact p-value) in the top 400 ORFs upregulated in each genotype.
(0.12 MB DOC)

Figure S1 Temporal Pearson correlation using MAS5 normalization. A) *Auto*- and B) *cross-correlations* for whole genome (22690 ORFs). C) *Auto*- and D) *cross-correlations* for immune-related genes.
(0.08 MB DOC)

Figure S2 Genome-wide expression changes between time points. Genome-wide expression changes (Δ*x*) between time points, 0–1 h (x-axis) vs. 1–4 h (y-axis) for groups of *N* ORFs (*N* = 10, 50, 80, 200) in A) wildtype, B) MyD88 KO, C) TRIF KO, D) and DKO. Group of n ORFs are sorted by their 0–1 h expression change (x-axis). Each point represents the average of Δ*x* for n ORFs. + and - indicate average of expression change of the upregulated and downregulated ORFs in each group, respectively.
(0.08 MB DOC)

Figure S3 Grouping of expression forms Gaussian distribution. Density distribution of all group of A) 50, B) 500 and C) 1000 ORFs sorted from highest to lowest for 0–1 h for each genotype. The density distribution of each of these groups in 1–4 h shows Gaussian distribution with decreasing fluctuations when group size increases (lighter color for increasing upregulated groups and darker color for increasing downregulated groups). x-axis represents Δ*x* for 1–4 h and y-axis represents the density of ORFs.
(0.11 MB DOC)

Figure S4 Genome-wide expression changes between genotypes. Genome-wide expression changes (Δ*x*) for 0–1 h between genotypes: A) wildtype vs. MyD88 KO, B) wildtype vs. TRIF KO, C) wildtype vs. DKO, D) TRIF KO vs. MyD88 KO, E) TRIF KO vs. DKO, F) MyD88 KO vs. DKO for groups of *N* ORFs (*N* = 10, 50, 80, 200). Group of *N* ORFs are sorted by their 0–1 h expression change (x-axis). Each point represents the average of Δ*x* for *N* ORFs. + and − indicate average of expression change of the upregulated and downregulated ORFs in each group.
(0.10 MB DOC)

Figure S5 Test for normality of genome-wide expression changes profiles. Average of *p*-values obtained from Shapiro-Wilk test for all groups of ORFs of wildtype collective mode in the MyD88 KO, TRIF KO, and DKO expression changes distribution when varying number of genes in the group.
(0.03 MB DOC)
