# Supplementary material for: Emergent Genome-Wide Control in Wildtype and Genetically Mutated Lipopolysaccarides-Stimulated Macrophages
Source: PLoS One. 2009 Mar 20;4(3):e4905. doi: 10.1371/journal.pone.0004905 (PMC2654147; doi:10.1371/journal.pone.0004905)
Supplement: Table S1 — List of immune-related genes. List of 157 immune-related genes selected from GenMAPP used for analysis. (0.16 MB DOC) [file pone.0004905.s001.doc]

**Table S1. List of immune-related genes**. 157 immune-related genes selected from GenMAPP

| **AFFY ID** | **Gene name** |
| --- | --- |
| 1415803_at | chemokine (c-x3-c motif) ligand 1 |
| 1416016_at | transporter 1, atp-binding cassette, sub-family b (mdr/tap) |
| 1416714_at | interferon regulatory factor 8 |
| 1416793_at | adp-ribosylation factor-like 6 interacting protein 2 |
| 1416847_s_at | 2'-5' oligoadenylate synthetase 1d/e |
| 1416978_at | fc receptor, igg, alpha chain transporter |
| 1417025_at | histocompatibility 2, class ii antigen e beta |
| 1417056_at | proteasome (prosome, macropain) 28 subunit, alpha |
| 1417266_at | chemokine (c-c motif) ligand 6 |
| 1417268_at | cd14 antigen |
| 1417574_at | chemokine (c-x-c motif) ligand 12 |
| 1417597_at | cd28 antigen |
| 1417705_at | otu domain, ubiquitin aldehyde binding 1 |
| 1417776_at | alpha-2-glycoprotein 1, zinc |
| 1417789_at | small chemokine (c-c motif) ligand 11 |
| 1417851_at | chemokine (c-x-c motif) ligand 13 |
| 1417898_a_at | granzyme a |
| 1417925_at | chemokine (c-c motif) ligand 22 |
| 1417932_at | interleukin 18 |
| 1417936_at | chemokine (c-c motif) ligand 9 |
| 1417948_s_at | interleukin enhancer binding factor 2 |
| 1418126_at | chemokine (c-c motif) ligand 5 |
| 1418131_at | sam domain and hd domain, 1 |
| 1418162_at | toll-like receptor 4 |
| 1418219_at | interleukin 15 |
| 1418265_s_at | interferon regulatory factor 2 |
| 1418293_at | interferon-induced protein with tetratricopeptide repeats 2 |
| 1418360_at | zinc finger protein 179 |
| 1418392_a_at | guanylate nucleotide binding protein 4 |
| 1418456_a_at | chemokine (c-x-c motif) ligand 14 |
| 1418480_at | chemokine (c-x-c motif) ligand 7 |
| 1418536_at | similar to mhc q8/9d surface antigen |
| 1418609_at | interleukin 1 family, member 6 |
| 1418638_at | histocompatibility 2, class ii, locus mb1 |
| 1418652_at | chemokine (c-x-c motif) ligand 9 |
| 1418685_at | toll-interleukin 1 receptor (tir) domain-containing adaptor protein |
| 1418686_at | 2'-5' oligoadenylate synthetase 1c |
| 1418718_at | chemokine (c-x-c motif) ligand 16 |
| 1418777_at | chemokine (c-c motif) ligand 25 |
| 1418803_a_at | fas ligand (tnf superfamily, member 6) |
| 1418930_at | chemokine (c-x-c motif) ligand 10 |
| 1419083_at | tumor necrosis factor (ligand) superfamily, member 11 |
| 1419132_at | toll-like receptor 2 |
| 1419135_at | lymphotoxin b |
| 1419209_at | chemokine (c-x-c motif) ligand 1 |
| 1419212_at | icos ligand |
| 1419272_at | myeloid differentiation primary response gene 88 |
| 1419282_at | chemokine (c-c motif) ligand 12 |
| 1419334_at | cytotoxic t-lymphocyte-associated protein 4 |
| 1419412_at | chemokine (c motif) ligand 1 |
| 1419426_s_at | chemokine (c-c motif) ligand 21a/b |
| 1419427_at | colony stimulating factor 3 (granulocyte) |
| 1419529_at | interleukin 23, alpha subunit p19 |
| 1419561_at | chemokine (c-c motif) ligand 3 |
| 1419596_at | ectodysplasin-a |
| 1419603_at | myeloid cell nuclear differentiation antigen/interferon activated gene 204/205 |
| 1419607_at | tumor necrosis factor |
| 1419684_at | chemokine (c-c motif) ligand 8 |
| 1419696_at | cd4 antigen |
| 1419697_at | chemokine (c-x-c motif) ligand 11 |
| 1419711_at | cd7 antigen |
| 1419728_at | chemokine (c-x-c motif) ligand 5 |
| 1419848_x_at | toll-like receptor 7 |
| 1420353_at | lymphotoxin a |
| 1420380_at | chemokine (c-c motif) ligand 2 |
| 1420412_at | tumor necrosis factor (ligand) superfamily, member 10 |
| 1420437_at | indoleamine-pyrrole 2,3 dioxygenase |
| 1420549_at | guanylate nucleotide binding protein 1 |
| 1420603_s_at | retinoic acid early transcript alpha/beta/delta/gamma |
| 1420782_at | tumor necrosis factor receptor superfamily, member 17 |
| 1420802_at | interleukin 13 |
| 1421034_a_at | interleukin 4 receptor, alpha |
| 1421104_at | macrophage activation 2 |
| 1421206_at | leukemia inhibitory factor |
| 1421228_at | chemokine (c-c motif) ligand 7 |
| 1421322_a_at | interferon dependent positive acting transcription factor 3 gamma |
| 1421352_at | toll-like receptor 6 |
| 1421358_at | histocompatibility 2, m region locus 3 |
| 1421370_a_at | interleukin 1 family, member 5 (delta) |
| 1421404_at | chemokine (c-x-c motif) ligand 15 |
| 1421473_at | interleukin 1 alpha |
| 1421547_at | cd180 antigen |
| 1421551_s_at | interferon activated gene 202 |
| 1421578_at | chemokine (c-c motif) ligand 4 |
| 1421588_at | tumor necrosis factor (ligand) superfamily, member 14 |
| 1421608_at | interleukin 20 |
| 1421638_at | carcinoembryonic antigen 2 |
| 1421688_a_at | chemokine (c-c motif) ligand 1 |
| 1421744_at | tumor necrosis factor (ligand) superfamily, member 4 |
| 1421898_a_at | major histocompatibility complex, class i-related |
| 1422005_at | eukaryotic translation initiation factor 2-alpha kinase 2 |
| 1422029_at | chemokine (c-c motif) ligand 20 |
| 1422080_at | interleukin 7 |
| 1422160_at | histocompatibility 2, t region locus 24 |
| 1422283_at | cd40 ligand |
| 1422416_s_at | pre-b lymphocyte gene 1/2 |
| 1422527_at | histocompatibility 2, class ii, locus dma |
| 1422645_at | hemochromatosis |
| 1422781_at | toll-like receptor 3 |
| 1422873_at | proteoglycan 2, bone marrow |
| 1422891_at | histocompatibility 2, class ii antigen e alpha |
| 1422903_at | lymphocyte antigen 86 |
| 1422924_at | tumor necrosis factor (ligand) superfamily, member 9 |
| 1422962_a_at | proteosome (prosome, macropain) subunit, beta type 8 (large multifunctional peptidase 7) |
| 1423017_a_at | interleukin 1 receptor antagonist |
| 1423047_at | toll interacting protein |
| 1424339_at | 2'-5' oligoadenylate synthetase-like 1 |
| 1424775_at | 2'-5' oligoadenylate synthetase 1a |
| 1424948_x_at | histocompatibility 2, k1, k region/mhc (a.ca/j(h-2k-f) class i antigen |
| 1425008_a_at | interferon activated gene 203 |
| 1425065_at | 2'-5' oligoadenylate synthetase 2 |
| 1425156_at | riken cdna 9830147j24 gene |
| 1425335_at | cd8 antigen, alpha chain |
| 1425374_at | 2'-5' oligoadenylate synthetase 3 |
| 1425454_a_at | interleukin 12a |
| 1425477_x_at | histocompatibility 2, class ii antigen a, beta 1 |
| 1425715_at | interleukin 1 family, member 8 |
| 1425925_at | fc receptor, iga, igm, high affinity |
| 1425958_at | interleukin 1 family, member 9 |
| 1426170_a_at | cd8 antigen, beta chain 1 |
| 1426181_a_at | interleukin 24 |
| 1427021_s_at | ferritin heavy chain 1/dipeptidase 2 |
| 1427429_at | colony stimulating factor 2 (granulocyte-macrophage) |
| 1435290_x_at | histocompatibility 2, class ii antigen a, alpha |
| 1435476_a_at | fc receptor, igg, low affinity iib |
| 1448436_a_at | interferon regulatory factor 1 |
| 1448995_at | chemokine (c-x-c motif) ligand 4 |
| 1449009_at | t-cell specific gtpase |
| 1449025_at | interferon-induced protein with tetratricopeptide repeats 3 |
| 1449049_at | toll-like receptor 1 |
| 1449184_at | peptidoglycan recognition protein 1 |
| 1449399_a_at | interleukin 1 beta |
| 1449508_at | interleukin 27 receptor, alpha |
| 1449556_at | histocompatibility 2, t region locus 23 |
| 1449874_at | lymphocyte antigen 96 |
| 1449875_s_at | histocompatibility 2, t region locus 9/10/22 |
| 1449924_at | proteoglycan 3 |
| 1449926_at | tumor necrosis factor (ligand) superfamily, member 7 |
| 1449984_at | chemokine (c-x-c motif) ligand 2 |
| 1449990_at | interleukin 2 |
| 1450242_at | toll-like receptor 5 |
| 1450272_at | tumor necrosis factor (ligand) superfamily, member 8 |
| 1450330_at | interleukin 10 |
| 1450488_at | chemokine (c-c motif) ligand 24 |
| 1450550_at | interleukin 5 |
| 1450565_at | interleukin 9 |
| 1450566_at | interleukin 3 |
| 1450587_at | histocompatibility 2, m region locus 10.1 |
| 1450696_at | proteosome (prosome, macropain) subunit, beta type 9 (large multifunctional peptidase 2) |
| 1450783_at | interferon-induced protein with tetratricopeptide repeats 1 |
| 1451644_a_at | histocompatibility 2, q region locus 1 |
| 1451957_at | interleukin 1 family, member 10 |
| 1452425_at | tumor necrosis factor receptor superfamily, member 14 (herpesvirus entry mediator) |
| 1452547_s_at | histocompatibility 2, d region locus 1/t region locus 18 |
| 1453196_a_at | 2'-5' oligoadenylate synthetase-like 2 |
| 1453913_a_at | transporter 2, atp-binding cassette, sub-family b (mdr/tap) |
| 1460251_at | fas (tnf receptor superfamily member) |
