# Supplementary material for: Emergent Genome-Wide Control in Wildtype and Genetically Mutated Lipopolysaccarides-Stimulated Macrophages
Source: PLoS One. 2009 Mar 20;4(3):e4905. doi: 10.1371/journal.pone.0004905 (PMC2654147; doi:10.1371/journal.pone.0004905)
Supplement: Table S2 — Differential activation of groups of ORFs between genotypes. Biological processes (Panther) and pathways (KEGG) enriched (p<0.05, Fisher-exact p-value) in the top 400 ORFs upregulated in each genotype. (0.12 MB DOC) [file pone.0004905.s002.doc]

**Table S2. Differential activation of groups of ORFs between genotypes. Biological processes (Panther) and *pathways* (KEGG) enriched (*p*<0.05, Fisher-exact *p*-value) in the top 400 ORFs upregulated in each genotype.**

| Location | **Wildtype** | | **MyD88 KO** | | **TRIF KO** | | **DKO** | |
| --- | --- | --- | --- | --- | --- | --- | --- | --- |
|  | Pathway | *p*-value | Pathway | *p*-value | Pathway | *p*-value | Pathway | *p*-value |
| Top 80* | *Cytokine-cytokine receptor interaction* | 1.2E-04 | *Type I diabetes mellitus* | 4.6E-04 | *MAPK signaling pathway* | 3.9E-05 | *p53 signaling pathway* | 1.3E-02 |
|  | *Toll-like receptor signaling pathway* | 4.9E-04 | *Cytokine-cytokine receptor interaction* | 2.9E-03 | *Toll-like receptor signaling pathway* | 7.2E-04 | *Cytokine-cytokine receptor interaction* | 3.7E-02 |
|  | *Apoptosis* | 1.6E-03 | *Apoptosis* | 1.2E-02 | *Cytokine-cytokine receptor interaction* | 1.3E-03 | T-cell mediated immunity | 3.2E-03 |
|  | *MAPK signaling pathway* | 5.2E-03 | *Hematopoietic cell lineage* | 1.6E-02 | *Apoptosis* | 2.2E-03 | MHCII-mediated immunity | 7.7E-03 |
|  | Signal transduction | 8.9E-04 | *Toll-like receptor signaling pathway* | 2.4E-02 | *T cell receptor signaling pathway* | 2.7E-02 | Other carbohydrate metabolism | 1.0E-02 |
|  | Proteolysis | 2.0E-02 | B-cell- and antibody-mediated immunity | 9.4E-03 | *Small cell lung cancer* | 2.8E-02 | Apoptosis | 3.2E-02 |
|  | Skeletal development | 2.7E-02 | Skeletal development | 1.4E-02 | *Cell Communication* | 4.9E-02 |  |  |
|  | Cell proliferation and differentiation | 2.7E-02 | Cell structure | 1.4E-02 | Proteolysis | 4.6E-04 |  |  |
|  | Stress response | 2.8E-02 | Immunity and defense | 2.6E-02 | Signal transduction | 6.4E-04 |  |  |
|  | Tricarboxylic acid pathway | 3.0E-02 | Signal transduction | 3.3E-02 | Nucleoside, nucleotide and nucleic acid metabolism | 5.6E-03 |  |  |
|  | Intracellular signaling cascade | 4.5E-02 | Vitamin metabolism | 4.3E-02 | tRNA metabolism | 1.4E-02 |  |  |
|  |  |  | MHCI-mediated immunity | 4.9E-02 | Cell proliferation and differentiation | 1.7E-02 |  |  |
|  |  |  |  |  | Intracellular signaling cascade | 2.6E-02 |  |  |
|  |  |  |  |  | Peroxisome transport | 2.9E-02 |  |  |
|  |  |  |  |  | Skeletal development | 3.7E-02 |  |  |
|  |  |  |  |  | Tricarboxylic acid pathway | 3.8E-02 |  |  |
|  |  |  |  |  | Other intracellular protein traffic | 4.6E-02 |  |  |
|  |  |  |  |  | Cell structure | 5.0E-02 |  |  |
| 80-160 | *ECM-receptor interaction* | 3.6E-02 | *B cell receptor signaling pathway* | 3.9E-02 | Vitamin metabolism | 1.2E-06 | Cell structure and motility | 1.9E-02 |
|  | *Small cell lung cancer* | 4.6E-02 | Cell motility | 5.8E-03 | Nucleoside, nucleotide and nucleic acid metabolism | 4.7E-04 |  |  |
|  | *Proteolysis* | 2.4E-03 | Sulfur metabolism | 2.8E-02 | RNA catabolism | 5.2E-03 |  |  |
|  | mRNA transcription regulation | 4.1E-03 | Cell structure and motility | 4.9E-02 | Vitamin biosynthesis | 5.6E-03 |  |  |
|  | Neurogenesis | 9.1E-03 |  |  |  |  |  |  |
|  | Cell structure and motility | 1.3E-02 |  |  |  |  |  |  |
|  | Oogenesis | 2.0E-02 |  |  |  |  |  |  |
|  | Ectoderm development | 2.1E-02 |  |  |  |  |  |  |
|  | mRNA transcription | 2.5E-02 |  |  |  |  |  |  |
|  | G-protein mediated signaling | 3.6E-02 |  |  |  |  |  |  |
| 160-240 | *B cell receptor signaling pathway* | 6.2E-03 | *Cell cycle* | 9.5E-03 | MHCI-mediated immunity | 7.6E-03 | *Chronic myeloid leukemia* | 1.0E-03 |
|  | Hematopoiesis | 1.4E-03 | Nucleoside, nucleotide and nucleic acid metabolism | 6.7E-03 | Nucleoside, nucleotide and nucleic acid metabolism | 1.2E-02 | Extracellular transport and import | 6.3E-03 |
|  | Proteolysis | 3.9E-02 | rRNA metabolism | 1.4E-02 | Apoptosis | 1.5E-02 | mRNA splicing | 2.9E-02 |
|  |  |  | Protein folding | 2.8E-02 |  |  | Lipid, fatty acid and steroid metabolism | 3.6E-02 |
|  |  |  | Gametogenesis | 3.3E-02 |  |  | G-protein mediated signaling | 4.6E-02 |
|  |  |  | Protein ADP-ribosylation | 3.3E-02 |  |  |  |  |
| 240-320 | *Apoptosis* | 1.6E-04 | Nucleoside, nucleotide and nucleic acid metabolism | 1.1E-02 | *Leukocyte transendothelial migration* | 5.4E-03 | *Insulin signaling pathway* | 4.8E-02 |
|  | *Toll-like receptor signaling pathway* | 6.0E-04 | Apoptosis | 2.0E-02 | *Gap junction* | 1.7E-02 | mRNA splicing | 7.2E-03 |
|  | *MAPK signaling pathway* | 6.4E-03 | Protein folding | 3.3E-02 | *MAPK signaling pathway* | 2.3E-02 | Pre-mRNA processing | 1.8E-02 |
|  | *B cell receptor signaling pathway* | 8.7E-03 | G-protein mediated signaling | 4.0E-02 | *Focal adhesion* | 3.6E-02 | Sulfur metabolism | 3.0E-02 |
|  | *Pancreatic cancer* | 1.3E-02 | Interferon-mediated immunity | 5.0E-02 | *Regulation of actin cytoskeleton* | 4.0E-02 |  |  |
|  | *Chronic myeloid leukemia* | 1.3E-02 |  |  | Cell communication | 8.5E-03 |  |  |
|  | *Jak-STAT signaling pathway* | 1.7E-02 |  |  | Intracellular protein traffic | 1.9E-02 |  |  |
|  | *T cell receptor signaling pathway* | 2.4E-02 |  |  | mRNA transcription regulation | 2.6E-02 |  |  |
|  | *Cytokine-cytokine receptor interaction* | 2.4E-02 |  |  | General vesicle transport | 3.3E-02 |  |  |
|  | T-cell mediated immunity | 9.7E-03 |  |  | Proteolysis | 3.3E-02 |  |  |
|  | mRNA transcription elongation | 9.8E-03 |  |  |  |  |  |  |
|  | mRNA transcription initiation | 3.9E-02 |  |  |  |  |  |  |
|  | Cell motility | 4.5E-02 |  |  |  |  |  |  |
| 320-400 | *Cytokine-cytokine receptor interaction* | *9.8E-03* | Sensory perception | 8.8E-03 | Vitamin biosynthesis | 1.1E-04 | Blood clotting | 1.8E-02 |
|  | *Jak-STAT signaling pathway* | *4.6E-02* | Antioxidation and free radical removal | 1.9E-02 | Vitamin metabolism | 5.0E-04 | DNA repair | 2.0E-02 |
|  | *MAPK signaling pathway* | *4.8E-02* | Phospholipid metabolism | 2.6E-02 | Coenzyme and prosthetic group metabolism | 1.3E-02 |  |  |
|  | mRNA transcription regulation | 1.7E-02 | Meiosis | 2.7E-02 | Antioxidation and free radical removal | 2.4E-02 |  |  |
|  | Chromosome segregation | 2.5E-02 | Constitutive exocytosis | 4.5E-02 | Protein biosynthesis | 3.0E-02 |  |  |
|  | Mesoderm development | 4.4E-02 | General vesicle transport | 4.6E-02 | Nucleoside, nucleotide and nucleic acid metabolism | 4.2E-02 |  |  |
|  | Cell cycle control | 4.5E-02 |  |  |  |  |  |  |
|  | Nucleoside, nucleotide and nucleic acid metabolism | 4.7E-02 |  |  |  |  |  |  |

* Acute mode for wildtype, MyD88 KO and TRIF KO.
