# Supplementary material for: Emergent Genome-Wide Control in Wildtype and Genetically Mutated Lipopolysaccarides-Stimulated Macrophages
Source: PLoS One. 2009 Mar 20;4(3):e4905. doi: 10.1371/journal.pone.0004905 (PMC2654147; doi:10.1371/journal.pone.0004905)

**Figure S1. Temporal Pearson correlation using MAS5 normalization**. A) *Auto*- and B) *cross*-*correlations* for whole genome (22690 ORFs). C) *Auto*- and D) *cross*-*correlations* for immune-related genes.


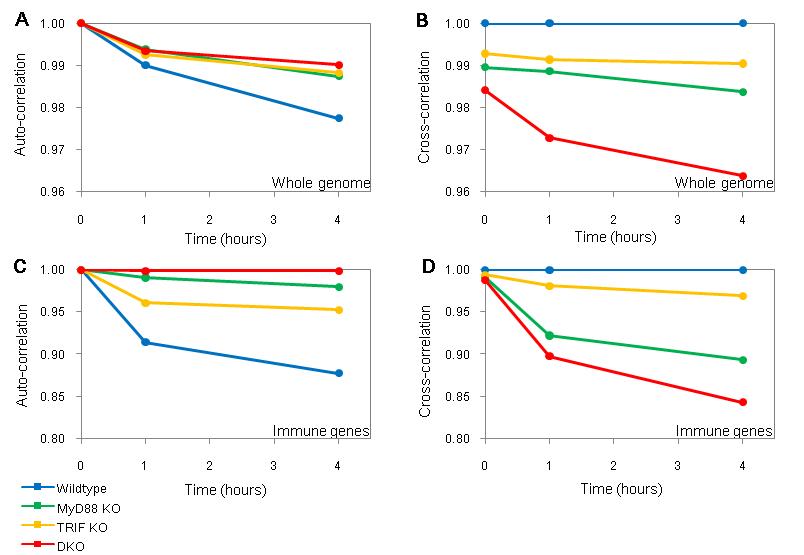

Supplement: Figure S1 — Temporal Pearson correlation using MAS5 normalization. A) Auto- and B) cross-correlations for whole genome (22690 ORFs). C) Auto- and D) cross-correlations for immune-related genes. (0.08 MB DOC) [file pone.0004905.s003.doc]
