# Supplementary material for: Emergent Genome-Wide Control in Wildtype and Genetically Mutated Lipopolysaccarides-Stimulated Macrophages
Source: PLoS One. 2009 Mar 20;4(3):e4905. doi: 10.1371/journal.pone.0004905 (PMC2654147; doi:10.1371/journal.pone.0004905)

**Figure S2. Genome-wide expression changes between time points.** Genome-wide expression changes (Δ*x*) between time points, 0-1h (x-axis) vs. 1-4h (y-axis) for groups of *N* ORFs (*N* =10, 50, 80, 200) in A) wildtype, B) MyD88 KO, C) TRIF KO, D) and DKO. Group of *N* ORFs are sorted by their 0-1h expression change (x-axis). Each point represents the average of Δ*x* for *N* ORFs. + and - indicate average of expression change of the upregulated and downregulated ORFs in each group, respectively.


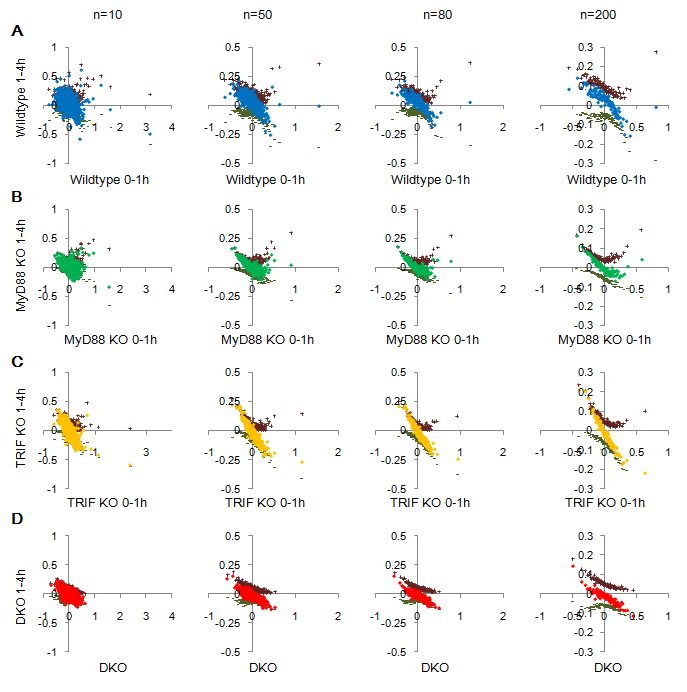

Supplement: Figure S2 — Genome-wide expression changes between time points. Genome-wide expression changes (Δx) between time points, 0–1 h (x-axis) vs. 1–4 h (y-axis) for groups of N ORFs (N = 10, 50, 80, 200) in A) wildtype, B) MyD88 KO, C) TRIF KO, D) and DKO. Group of n ORFs are sorted by their 0–1 h expression change (x-axis). Each point represents the average of Δx for n ORFs. + and - indicate average of expression change of the upregulated and downregulated ORFs in each group, respectively. (0.08 MB DOC) [file pone.0004905.s004.doc]
