# Supplementary material for: Emergent Genome-Wide Control in Wildtype and Genetically Mutated Lipopolysaccarides-Stimulated Macrophages
Source: PLoS One. 2009 Mar 20;4(3):e4905. doi: 10.1371/journal.pone.0004905 (PMC2654147; doi:10.1371/journal.pone.0004905)

**Figure S3. Grouping of expression forms Gaussian distribution.** Density distribution of all group of A) 50, B) 500 and C) 1000 ORFs sorted from highest to lowest for 0-1h for each genotype. The density distribution of each of these groups in 1-4h shows Gaussian distribution with decreasing fluctuations when group size increases (lighter color for increasing upregulated groups and darker color for increasing downregulated groups). x-axis represents Δ*x* for 1-4h and y-axis represents the density of ORFs.


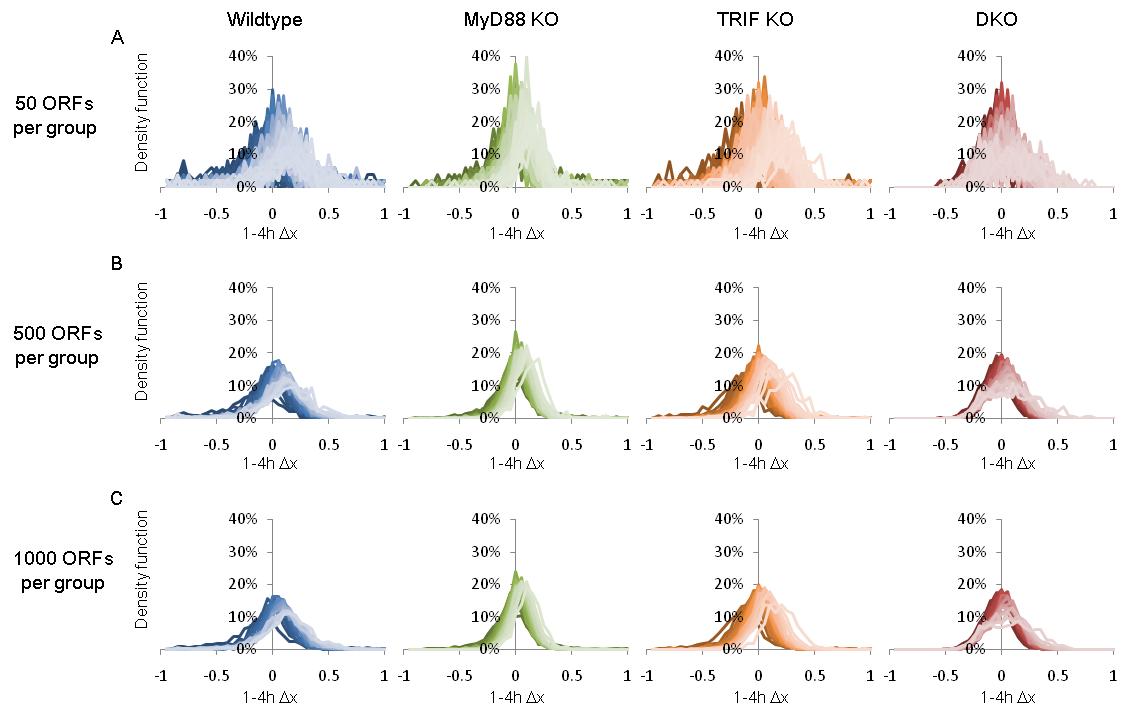

Supplement: Figure S3 — Grouping of expression forms Gaussian distribution. Density distribution of all group of A) 50, B) 500 and C) 1000 ORFs sorted from highest to lowest for 0–1 h for each genotype. The density distribution of each of these groups in 1–4 h shows Gaussian distribution with decreasing fluctuations when group size increases (lighter color for increasing upregulated groups and darker color for increasing downregulated groups). x-axis represents Δx for 1–4 h and y-axis represents the density of ORFs. (0.11 MB DOC) [file pone.0004905.s005.doc]
