# Supplementary material for: Emergent Genome-Wide Control in Wildtype and Genetically Mutated Lipopolysaccarides-Stimulated Macrophages
Source: PLoS One. 2009 Mar 20;4(3):e4905. doi: 10.1371/journal.pone.0004905 (PMC2654147; doi:10.1371/journal.pone.0004905)

**Figure S4. Genome-wide expression changes between genotype.** Genome-wide expression changes (Δ*x*) for 0-1h between genotypes: A) wildtype vs. MyD88 KO, B) wildtype vs. TRIF KO, C) wildtype vs. DKO, D) TRIF KO vs. MyD88 KO, E) TRIF KO vs. DKO, F) MyD88 KO vs. DKO for groups of *N* ORFs (*N*=10, 50, 80, 200). Group of *N* ORFs are sorted by their 0-1h expression change (x-axis). Each point represents the average of Δ*x* for *N* ORFs. + and - indicate average of expression change of the upregulated and downregulated ORFs in each group.


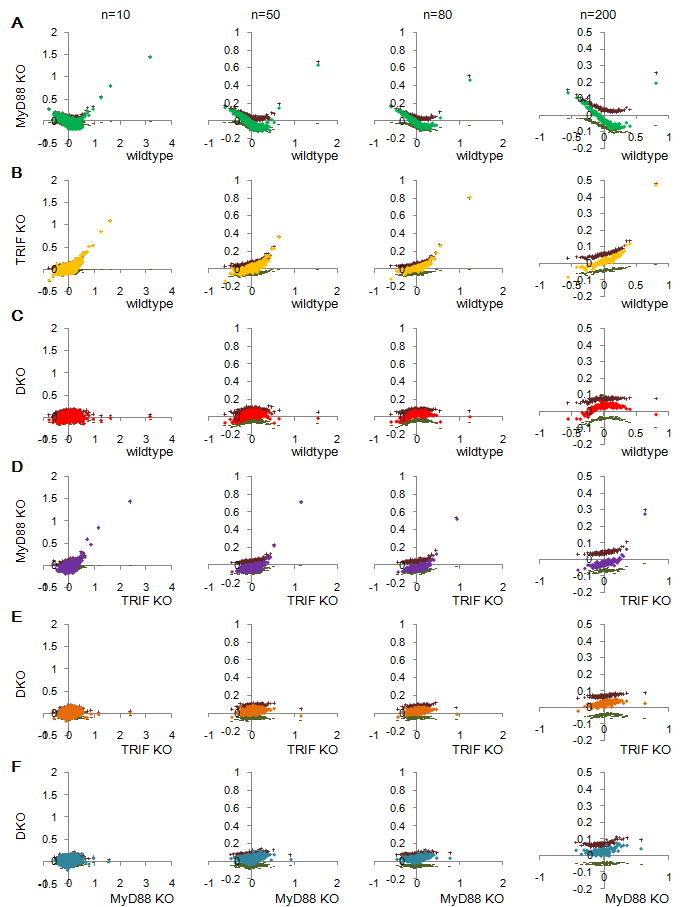

Supplement: Figure S4 — Genome-wide expression changes between genotypes. Genome-wide expression changes (Δx) for 0–1 h between genotypes: A) wildtype vs. MyD88 KO, B) wildtype vs. TRIF KO, C) wildtype vs. DKO, D) TRIF KO vs. MyD88 KO, E) TRIF KO vs. DKO, F) MyD88 KO vs. DKO for groups of N ORFs (N = 10, 50, 80, 200). Group of N ORFs are sorted by their 0–1 h expression change (x-axis). Each point represents the average of Δx for N ORFs. + and − indicate average of expression change of the upregulated and downregulated ORFs in each group. (0.10 MB DOC) [file pone.0004905.s006.doc]
