# Supplementary material for: Emergent Genome-Wide Control in Wildtype and Genetically Mutated Lipopolysaccarides-Stimulated Macrophages
Source: PLoS One. 2009 Mar 20;4(3):e4905. doi: 10.1371/journal.pone.0004905 (PMC2654147; doi:10.1371/journal.pone.0004905)

**Figure S5. Test for normality of genome-wide expression changes profiles.** Average of *p-*values obtained from Shapiro-Wilk test for all groups of ORFs of wildtype *collective* mode in the MyD88 KO, TRIF KO, and DKO expression changes distribution when varying number of genes in the group.


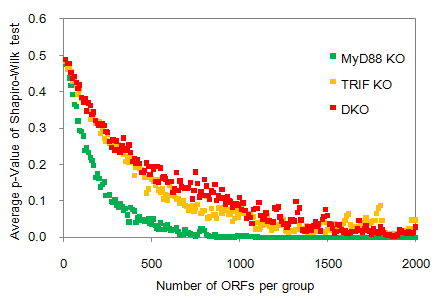

Supplement: Figure S5 — Test for normality of genome-wide expression changes profiles. Average of p-values obtained from Shapiro-Wilk test for all groups of ORFs of wildtype collective mode in the MyD88 KO, TRIF KO, and DKO expression changes distribution when varying number of genes in the group. (0.03 MB DOC) [file pone.0004905.s007.doc]
